# Managerial attitude to the implementation of quality management systems in Lithuanian support treatment and nursing hospitals

**DOI:** 10.1186/1472-6963-6-120

**Published:** 2006-09-20

**Authors:** Ilona Buciuniene, Sonata Malciankina, Zigmas Lydeka, Ruta Kazlauskaite

**Affiliations:** 1 Department of Intellectual Capital and Business Competence, ISM University of Management and Economics, Ozeskienes Street 18, LT-44254 Kaunas, Lithuania; 2 Department of Social Medicine, Kaunas University of Medicine, Kaunas, Lithuania; 3 Kaunas Terminal Care Hospital, Kaunas, Lithuania; 4 Vytautas Magnus University, Kaunas, Lithuania

## Abstract

**Background:**

The regulations of the Quality Management System (QMS) implementation in health care organizations were approved by the Lithuanian Ministry of Health in 1998. Following the above regulations, general managers of health care organizations had to initiate the QMS implementation in hospitals. As no research on the QMS implementation has been carried out in Lithuanian support treatment and nursing hospitals since, the objective of this study is to assess its current stage from a managerial perspective.

**Methods:**

A questionnaire survey of general managers of Lithuanian support treatment and nursing hospitals was carried out in the period of January through March 2005. Majority of the items included in the questionnaire were measured on a seven-point Likert scale. During the survey, a total of 72 questionnaires was distributed, out of which 58 filled-in ones were returned (response rate 80.6 per cent; standard sampling error 0.029 at 95 per cent level of confidence).

**Results:**

Quality Management Systems were found operating in 39.7 per cent of support treatment and nursing hospitals and currently under implementation in 46.6 per cent of hospitals (13.7% still do not have it). The mean of the respondents' perceived QMS significance is 5.8 (on a seven-point scale). The most critical issues related to the QMS implementation include procedure development (5.5), lack of financial resources (5.4) and information (5.1), and development of work guidelines (4.6), while improved responsibility and power sharing (5.2), better service quality (5.1) and higher patient satisfaction (5.1) were perceived by the respondents as the key QMS benefits. The level of satisfaction with the QMS among the management of the surveyed hospitals is mediocre (3.6). However it was found to be higher among respondents who were more competent in quality management, were familiar with ISO 9000 standards, and had higher numbers of employees trained in quality management.

**Conclusion:**

QMSs are perceived to be successfully running in one third of the Lithuanian support treatment and nursing hospitals. Its current implementation stage is dependent on the hospital size – the bigger the hospital the more success it meets in the QMS implementation. As to critical Quality Management (QM) issues, hospitals tend to encounter such major problems as lack of financial resources, information and training, as well as difficulties in procedure development. On the other hand, the key factors that assist to the success of the QMS implementation comprise managerial awareness of the QMS significance and the existence of employee training systems and audit groups in hospitals.

## Background

In the 1990s, the greater part of the world's health care policy makers realised the great significance of quality in health care organizations, which led to the initiation of respective actions. For instance, Swedish hospitals started quality-related initiatives back in 1992 [1]. France started its health care reform in 1996, which among other issues was aimed at quality assurance and hospital accreditation [2]. Considerable attention has recently been given to the effective management and delivery of health care services in Irish health care organizations [3]. The 1997 UK government White Paper clearly stated that sustaining of the NHS was reliant upon a "new drive for quality" [4]. In the Netherlands, a law passed in 1996 provided national quality requirements for health care organizations [5].

The Quality Management System (QMS) is a coordinated aggregate of interrelated and interactive activities that determine quality policy and objectives as well as provides health care organisations with guidance and rules in their goal attainment [6]. The implementation of quality management systems enables health care organizations to define and manage processes that ensure delivery of services that meet customer needs and expectations. Besides it installs trust in both organizations and consumers in respect to service quality and conformity to respective standards.

Lithuanian health care organizations are obliged to implement quality management systems as to the law passed by the Lithuanian Ministry of Health in October 1998 [7]. The above law provides the procedure of the QMS implementation in health care organizations and commits their management to its execution: the general manager of a hospital has to appoint the local audit group manager, who in turn is responsible for the formation of the audit group. The latter then controls the QMS operation, prepares all the necessary documentation, etc. However the Ministry of Health did not provide health care organizations the needed support in QMS implementation and did not ensure a systemic coordination of the quality improvement process. It was only in 2000, when in collaboration with the Danish Health Ministry, guidelines for QMS implementation were developed and published [8].

The QMS implementation encountered a number of barriers in Lithuania, such as insufficient methodological assistance from respective state institutions, lack of quality management knowledge, insufficient motivation of health care managers and professionals to participate in health care quality improvement activities, and quality standards [8], which conditioned slow national development of the QMS in health care organizations.

In the Soviet period (1941–1990), Lithuania did not have any support treatment and nursing hospitals. The first ones were established in the country only in 1990, after the initiation of the national health care reform. As to Lithuanian medical norm MN 80:2000, support treatment and nursing hospitals are health care organizations that provide first level in-patient support treatment and nursing services to disabled persons and people suffering from chronic ailments [9]. The above norm defines support treatment as in-patient medical support provided to patients for whom diagnosis has been made and who do not require specialized treatment [9]. Nursing is defined as nursing activities that assist in sustaining a patient's vital body functions [9]. Support treatment and nursing hospitals provide long-term treatment, i.e. patients are hospitalised for up to 120 and more days, which necessitates assuring qualitative care and meeting patients' needs. Lithuanian support treatment and nursing hospitals have to follow the above-mentioned general regulations of the QMS implementation.

### Research objective

As no research on the QMS implementation has been carried out in Lithuanian support treatment and nursing hospitals before, the objective of this study is to assess its current QMS implementation stage from a managerial perspective.

#### Research goals

1. to examine managerial perceptions of the current QMS implementation progress in Lithuanian support treatment and nursing hospitals;

2. to identify problems arising in the QMS implementation in Lithuanian support treatment and nursing hospitals;

3. to assess the level of managerial satisfaction with the currently operating quality management systems;

4. to compare and contrast the QMS implementation in different size hospitals.

## Methods

### Sample

A population survey of Lithuanian support treatment and nursing hospitals was carried out. According to the data of the Lithuanian Health Information Centre, there were 72 hospitals of the kind in Lithuania in 2004 [10]. The study group consisted of the management of support treatment and nursing hospitals.

A quantitative research method, i.e. a questionnaire survey, was chosen to attain the research goals. The survey was a cross sectional, one time assessment of the managerial attitude to the QMS implementation in support treatment and nursing hospitals. The survey was carried out in the period of January through March 2005. During the survey, a total of 72 questionnaires was distributed, out of which 58 filled-in ones were returned (response rate 80.6 per cent; standard sampling error 0.029 at 95 per cent level of confidence) [11].

### Questionnaire development

Given the fact that the regulations of the QMS implementation in Lithuanian health care organizations were developed under ISO standards [6], the questionnaire was designed following the above standards and prior research on QMS in health care organizations [12-18].

The questionnaire items fell under the following four major categories: 1) general questions, 2) questions on the current QMS implementation stage, 3) questions on problems that may have arisen in the QMS implementation process, and 4) questions on QMS benefits and the level of managerial satisfaction with the operating QMS. The respondents were asked to rank their agreement/disagreement with the provided statements on a seven-point Likert scale (see Additional file 1).

### Questionnaire reliability

Cronbach's Alpha was used to assess the questionnaire reliability (only for items measured on a seven-point Likert scale). An alpha value of 0.80 or higher was considered as acceptable reliability [19]. The questionnaire reliability coefficient was 0.86. The questionnaire was pilot tested with ten managers of randomly selected hospitals.

### Data collection process

Questionnaire packages contained a cover letter with an explanation of the survey purpose and guarantee of response confidentiality, a questionnaire, and a pre-paid return envelope. Questionnaires were mailed to general managers of 72 support treatment and nursing hospitals. After a week, all the above managers were telephoned and reminded of the survey. 20 filled-in questionnaires were received after the first call. In another two weeks, the remaining 52 hospitals were given a second call. All in all, 58 filled-in questionnaires were returned.

### Data analysis

The survey data was processed using the SPSS statistical package (version 11). The statistical data reliability was checked according to χ^2 ^criteria, degrees of freedom number (df), and statistical significance. The relationship between two independent variables was assessed by calculating Spearman's rank correlation coefficients, taking into consideration the value of the correlation ratio and its statistical significance (reliability notation: p < 0.05 means statistically significant, p < 0.01 highly significant). Explanatory factor analysis was used to reduce the number of QMS-related benefits and problems.

For analysis purposes, all support treatment and nursing hospitals under the survey were subdivided into three major groups in respect to the number of employees:

1. *small*, with the employee number under 50;

2. *medium*, with employee number ranging between 50 and 100;

3. *large*, with employee number over 100.

Respectively 46.6 per cent of the support treatment and nursing hospitals fell under the first group, 29.3 were categorized as medium sized, and 24.1 small-sized (see Table 1). Based on the number of years spent working for a health care organization, respondents distributed as follows: 29 (50%) have worked for up to 5 years; 23 (40%) 6 to 10 years; and 6 (10%) over 10 years. The above rather low figures can be accounted for by the fact that the first support treatment and nursing hospitals were established only in 1990.

## Results

### The current QMS implementation stage: managerial perceptions

The analysis of managerial attitude towards the QMS implementation in Lithuanian support treatment and nursing hospitals showed that the QMS operates in 39.7 per cent of organizations and is currently under development in 46.6 per cent of hospitals (13.7 per cent still do not have it). The QMS implementation stage was found to vary among different size hospitals: it is under development in 51.9 per cent of small, 58.8 per cent of medium, and 21.4 per cent of large hospitals (see Table 1).

Quality audit groups have been set-up in 70.7 per cent of hospitals, while 29.3 per cent of organizations still do not have them. It is noteworthy that all large-scale hospitals have audit groups, while the same figure among small size institutions is just a bit over 50 per cent.

Employee training systems currently operate in 56.9 per cent of hospitals. QM-related training systems have been developed in a statistically significant greater part of large hospitals (85.7%) in comparison to medium (58.8%) and small-size (40.7%) hospitals.

### Perceived QMS significance

The mean of the perceived QMS significance is 5.76 on a seven-point scale (see Table 2). In respect to the hospital size, there are no statistically significant differences in the QMS significance ranking among the three hospital groups under this study. However, the ranking of the perceived QMS significance differs between hospitals with QM training systems (mean = 6.09) and hospitals that do not have such systems (5.23) (p < 0.05).

**Figure 1:**
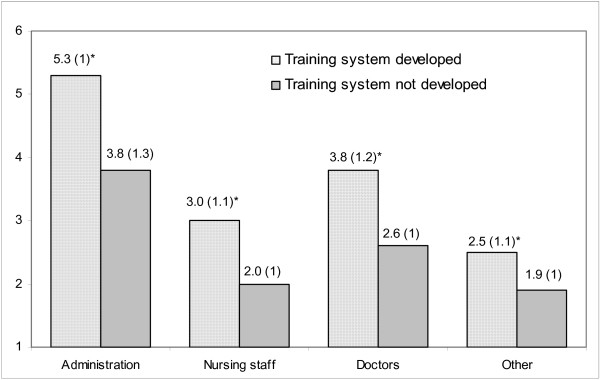
Mean score of perceived employee quality management competence in respect to the existence of respective training systems (standard deviation).

Depending on the existence/absence of employee training systems on quality management, research brought out a statistically significant difference as regards the managerial assessment of employee knowledge, i.e. managers of hospitals that have training systems rank their employee knowledge higher than those that do not have them (see Figure 1).

### Satisfaction with QMS

The overall managerial satisfaction with the QMS is barely moderate (mean = 3.6 on a 7 point scale) (see Table 2). Research findings show some statistically significant variances in satisfaction rating among different size hospitals: managers of large hospitals tend to be more satisfied with the QMS (4.5) than their counterparts in medium (3.8) and small (3.0) hospitals. Besides research results revealed statistically significant higher levels of managerial satisfaction with QMS in hospitals that have developed QM training systems (6.09) in contrast to those that have not (5.76).

Managerial satisfaction with the QMS was found to bear a high degree correlation with QM knowledge (Spearman's correlation ratio = 0.754, p < 0.01) and staff training in QM (0.708, p < 0.01) (see Table 3). Moderate degrees of correlation were found between the managerial satisfaction with the QMS and knowledge of ISO 9000 standards (0.623, p < 0.01), doctors' QM competence (0.617, p < 0.01), administration's QM competence (0.548, p < 0.01), and nursing staff QM competence (0.540, p < 0.01).

### QMS implementation problems: managerial perceptions

Major problems that hospitals encountered in the QMS implementation tend to be as follows: procedure development (mean = 5.45), lack of financial resources (5.38), lack of information (5.06), and development of work instructions (4.73) (see Table 4). Some variances in the rating of the critical QMS implementation issues among different size hospitals were detected, but they are not statistically significant.

The results of the factor analysis of the QMS implementation problems indicate that there are four rotated factors, which explained a total variance of 73.26 per cent (KMO MSA = 0.557, χ^2 ^= 252.300, df = 55, p = 0.000). These are as follows: *QM competence and organizational resources *(staff training, procedure development, development of work instructions, lack of information, and lack of financial resources), *QM policy *(quality policy definition and quality goal setting), *QM audit group activities *(appointment of local audit group manager, audit group formation, and use of diagnostic and treatment methods), and *staff resistance to the QMS implementation *(see Table 5).

### Perceived QMS organizational benefits

**Figure 2:**
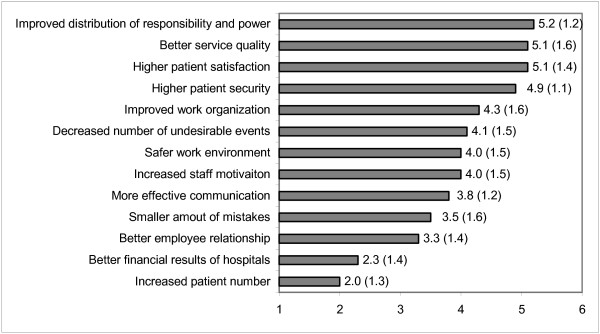
Mean score of QMS perceived benefit ranking (standard deviation).

Out of the 13 proposed QMS organizational benefits, hospital managers perceived the following ones as of the highest relevance to their hospitals: improvement of responsibility and power sharing (mean = 5.2), better service quality (5.1), higher patient satisfaction (5.1), and a stronger sense of security among patients (4.9) (see Figure 2).

Research findings showed that the relationships between the perceived QMS significance and 11 organizational benefits are statistically significant; the above relationships, however, are low to moderate (see Table 6). The highest of those were found between the perceived QMS significance and higher patient satisfaction (Spearman's ratio = 0.577, p < 0.01), more effective communication (0.477, p < 0.01), safer work environment (0.461, p < 0.01), and improved work organization (0.402, p < 0.01).

The results of the factor analysis of the perceived QMS organizational benefits show that there are three rotated factors, which explained a total variance of 63.64 per cent (KMO MSA = 0.824, χ^2 ^= 307.555, df = 78, p = 0.000). These are as follows: *improved work quality and safety *(a stronger sense of security among patients, a lower number of undesirable incidents, a lower number of mistakes, improved work organization, and safer work environment), *improved service quality and patient/employee satisfaction *(improved service quality, higher patient satisfaction, more effective communication, higher employee motivation, improved responsibility and authority sharing), and *improved organizational performance *(improved financial situation, better employee relationship, and increased patient number) (see Table 7).

## Discussion

Research findings showed that given the managerial perceptions the QMS has so far been implemented in one third of Lithuanian support treatment and nursing hospitals. Prior research on the QMS implementation in another three EU countries revealed less prominent results: the QMS was implemented in 4 per cent of Dutch hospitals, 0 per cent in Hungary, and 3 per cent in Finland [5]. However this comparison does not allow making any far-reaching conclusions, as the two studies used different QMS implementation assessment measures. The Lithuanian study was based on the managerial perceptions of the QMS implementation, and the three-country research focused on a number of QM activities based on which the QMS implementation stage was determined. Besides it was carried out in general hospitals, while the Lithuanian case is exclusively built on the support treatment and nursing hospitals.

The biggest contribution of this research is that it shows a relationship between the hospital size and QMS implementation. This issue has not been studied earlier, yet it is one of the most crucial factors in the QMS implementation in hospitals. Though, on the one hand, the scope of QMS-related work is typically narrower in small organizations in comparison to large ones, on the other hand, smaller organizations usually suffer a bigger shortage of resources, especially human resources. Such a situation, in turn, conditions problems in the establishment of audit groups, which are one of the key success factors in the QMS implementation. Larger institutions have fewer problems, and their management experience higher levels of satisfaction with the QMS. Small hospitals are therefore recommended to cooperate in the establishment of audit groups, i.e. forming one audit group which would comprise representatives delegated by a number of hospitals. This would assist in solving the problem of human resource scarcity and facilitate procedure development. It is also recommended for smaller hospitals to share experience in staff involvement in QM processes, and cooperate in organising staff training in QM.

From the managerial perspective, the most critical issues encountered in the QMS implementation in Lithuanian support treatment and nursing institutions were as follows: procedure development, personnel training, and lack financial resources and information. The above research findings are congruent to prior study results, which also point out such QMS-related problems as lack of financial resources [13,14] and HRM-related critical issues [14].

Taking into account the fact that QMS implementation was initiated in Lithuanian hospitals at the end of 1998 and a relatively small size of support treatment and nursing hospitals, it can be stated that the QMS implementation in the above institutions is rather lengthy (7 years) in comparison to business organizations, where QMS implementation averages 1.67 years [20]. An assumption thus can be made that such a slow QMS implementation process is related to a lack of comprehensive standards authorised by the Ministry of Health [12]. The paper, however, did not look into the key factors that might have conditioned this rather lengthy time scope. However, the above situation may possibly be accounted for by the inefficiency of the national health care policy, insufficient commitment to QM, etc. Whatever the causes, the lengthy QMS implementation constitutes a serious problem and calls for an adequate consideration on the side of health care politicians.

Research into the implementation of quality management systems in Lithuanian support treatment and nursing hospitals supported the previously determined key success factor of QMS implementation, i.e. managerial perception of the QMS significance [1,21]. Findings of the present research show that managerial perception of the QMS significance correlates with certain QMS organizational benefits, such as higher patient satisfaction, more effective communication, safer work environment, better work organization, etc. This relationship might be explained by the fact that the managerial attitude to QM conditions key QMS-related decisions, such as resource allocation and development of QM training systems, which in turn leads to higher levels of QM competence among all level employees.

Managerial satisfaction with the QMS is associated with the level of their QM knowledge as well as their staff training in quality management. Thus QM training of management and staff conditions the success of the QMS implementation in hospitals. In parallel to prior studies, this research also showed that appropriate training is highly significant in the QMS implementation [21,22].

As to the most valued benefits of the QMS (as perceived by hospital managers), these include improved responsibility and power sharing, better service quality, higher patient satisfaction with services, and improved work organisation. The above research findings are parallel to the results of prior study in health care, which among other benefits comprise patient satisfaction, excellent professional outcomes, no delays, etc. [15].

### Limitations

The paper did not attempt to determine the actual stage of the QMS implementation, assessment of which would have necessitated such means as quality audit, benchmarking, etc. The above issue could serve as a research object for further studies in the area. This paper is built on the managerial perceptions of the QMS implementation, which may bear a certain level of subjectivity.

## Conclusion

Research findings show that as perceived by hospital managers QMS operates in more than a third of Lithuanian support treatment and nursing hospitals. QMS implementation is dependant on the hospital size – currently QMS operates in the majority of large hospitals (over 100 employees), whereas in about half of smaller hospitals QMS is still under implementation.

From the managerial perspective, the most critical issues encountered in the QMS implementation in Lithuanian support treatment and nursing institutions were as follows: procedure development and scarcity of financial resources and information.

Heads of Lithuanian support treatment and nursing hospital consider QMS implementation highly relevant for their hospitals. More than fifty per cent of hospitals have established employee-training systems, and heads of such institutions tend to rate their employee quality management competence (skills and abilities) considerably higher than those from hospitals that do not have respective training systems.

The overall level of managerial satisfaction with the operating QMS is merely mediocre. As to the most valued benefits as perceived by hospital heads these include improved allocation of responsibility and power, service quality, and patient satisfaction with services.

## Competing interests

The author(s) declare that they have no competing interests.

## Authors' contributions

These authors equally contributed to this work.

## Pre-publication history

The pre-publication history for this paper can be accessed here:



## Supplementary Material

Additional File 1 Survey questionnaire items. Click here for file

## Figures and Tables

**Table 1:** Current stage of quality system implementation in Lithuanian support treatment and nursing hospitals in respect to the hospital size

**Criterion**	**Hospital size (number of employees)**						**Total**		
				
	Small (Under 50)		Medium (50–100)		Large (over 100)				
		
	n	%	n	%	n	%	n	%	
**Current QMS implementation stage**									χ^2 ^= 15.69; df = 4; p = 0.003. * p < 0.05 as compared to large-size hospitals. ** p < 0.05 in comparison to other size hospitals
QMS under implementation	14	51.9	10	58.8	3	21.4*	27	46.6	
QMS implemented	6	22.2	6	35.3	11	78.6**	23	39.7	
QMS implementation not started	7	25.9	1	5.9	0	0.0	8	13.8	
Total	27	100	17	100	14	100	58	100	
**Quality audit group formation**									χ^2 ^= 10.7; df = 2; p = 0.005; * p < 0.05 in comparison to other size hospitals having audit groups.
Group formed	14	51.9*	13	76.5	14	100	41	70.7	
Group not formed	13	48.1	4	23.5	0	0	17	29.3	
Total	27	100	17	100	14	100	58	100	
**Training in QMS**									χ^2 ^= 7.64; df = 2; p = 0.02.
System developed	11	40.7	10	58.8	12	85.7	33	56.9	
System absent	16	59.3	7	41.2	2	14.3	25	43.1	
Total	27	100	17	100	14	100	58	100	

**Table 2:** Perceived QMS significance and satisfaction with QMS in Lithuanian support treatment and nursing hospitals

**Criterion**	**QMS significance**		**Satisfaction with QMS**		
		
	**Means**	**Standard deviation (SD)**	**Means**	**Standard deviation (SD)**	
Overall	5.76	1.14	3.6	1.6	
Hospitals with quality management training systems	6.09*	1.07	4.3*	1.1	* p < 0.05, in comparison to hospitals that do not have training systems
Hospitals without quality management training systems	5.23	1.11	2.6	1.6	
Hospitals with < 50 employees	5.78	1.01	3.0	1.8	* p < 0.05, in comparison to other size hospitals
Hospitals with 50 to 100 employees	5.47	1.23	3.8	1.1	
Hospitals with >100 employees	6.07	1.27	4.5*	1.2	

**Table 3:** Relationship between satisfaction with QMS and criterion affecting it

**Criterion**		**Satisfaction with QMS (Spearman's ratio)**
Respondent's knowledge of quality management		.754**
Respondent's knowledge of ISO 9000 standards		.623**
Proportion of hospital staff to have attended training in quality management		.708**
Competence in quality management among other hospital staff (as perceived by the respondents)	Administration	.548**
	Nursing staff	.540**
	Doctors	.617**
	Others	.383**

** p < 0.01.

**Table 4:** QMS implementation problems in Lithuanian support treatment and nursing hospitals

**QMS implementation problem**	**Hospital size (number of employees)**						**General**				
						
	**Small (under 50)**		**Medium (50–100)**		**Large (over 100)**						
	
	**Mean**	**SD**	**Mean**	**SD**	**Mean**	**SD**	**Mean**	**SD**	**X**^2^	**df**	**p**
Quality policy definition	2.55	1.57	2.47	1.12	2.86	1.56	2.60	1.42	0.60	2	.74
Quality goal setting	2.86	1.52	2.59	1.12	2.64	1.78	2.72	1.46	0.72	2	.70
Appointment of local audit group manager	3.14	1.61	3.29	1.69	2.71	1.59	3.08	1.62	1.17	2	.56
Audit group formation	4.68	2.15	4.18	1.91	3.07	1.64	4.09	2.02	5.77	2	.06
Personnel training	4.18	1.18	4.47	1.42	4.57	1.50	4.38	1.33	1.09	2	.58
Procedure development	5.41	1.44	5.65	1.37	5.29	1.44	5.45	1.39	0.55	2	.76
Development of job instructions	4.73	1.45	4.65	1.00	4.50	1.87	4.64	1.43	0.06	2	.97
Use of diagnostic and treatment methods	3.91	1.41	3.82	1.55	4.29	1.54	3.98	1.47	0.55	2	.76
Staff resistance to QMS implementation	3.09	1.48	2.88	1.05	2.64	1.50	2.91	1.35	1.26	2	.53
Information deficiency	4.68	1.36	5.65*	1.41	4.93	1.49	5.06	1.45	6.46	2	.04
Lack of financial resources	5.32	1.81	5.71	1.31	5.07	1.86	5.38	1.67	0.66	2	.72

* p < 0.05, statistically significant difference in comparison to hospitals with employee number under 50

**Table 5:** Factor analysis of the perceived QMS benefits

**Factor**	**Variable**	**Loading**	**Total variance explained**
Factor I **Improved work quality and safety**	Stronger sense of security among patients	.727	44.45%
	Lower number of undesirable incidents	.782	
	Lower number of mistakes	.673	
	Improved work organization	.548	
	Safer work environment	.760	
Factor II **Improved service quality and patient/employee satisfaction**	Improved service quality	.736	10.43%
	Higher patient satisfaction	.630	
	More effective communication	.611	
	Higher employee motivation	.695	
	Improved responsibility and authority sharing	.498	
Factor III **Improved organizational performance**	Improved financial situation	.466	8.76%
	Better employee relationship	.707	
	Increased patient number	.441	

**Table 6:** Spearman's correlation ratio between perceived QMS organizational benefits and QMS significance

**QMS benefit**	**QMS significance (Spearman's ratio)**
Improved financial situation	.386**
Higher service quality	.349*
Higher employee motivation	.379**
Stronger sense of security among patients	.332*
Lower number of mistakes	.321*
Better employee relationship	.377**
Lower number of undesirable incidents	.389**
Improved work organization	.402**
Higher patient satisfaction	.577**
More effective communication	.477**
Safer work environment	.461**
Increased patient number	.2
Improved responsibility and authority sharing	.26

* p < 0.05; ** p < 0.01

**Table 7:** Factor analysis of the QMS implementation problems

**Factor**	**Variable**	**Loading**	**Total variance explained**
**Factor I (QM competence and organizational resources)**	Staff training	.432	25.24%
	Procedure development,	.833	
	Development of work instructions	.709	
	Lack of information	.670	
	Lack of financial resources	.642	
**Factor II (QM policy)**	Quality policy definition	.891	22.53%
	Quality goal setting	.893	
**Factor III (QM audit group activities)**	Appointment of local audit group manager	.679	15.13%
	Audit group formation	.740	
	Use of diagnostic and treatment methods	.432	
**Factor IV (Employee resistance to QMS implementation)**	Employee resistance to QMS implementation	.854	10.35%
