# Supplementary material for: Managerial attitude to the implementation of quality management systems in Lithuanian support treatment and nursing hospitals
Source: BMC Health Serv Res. 2006 Sep 20;6:120. doi: 10.1186/1472-6963-6-120 (PMC1592079; doi:10.1186/1472-6963-6-120)
Supplement: Additional File 1 — Survey questionnaire items. [file 1472-6963-6-120-S1.doc]

**Questionnaire items**

**General questions**

- number of employees (please specify);
- number of years spent working for a health care institution (please specify);

**Questions on current QMS implementation stage**

- knowledge of quality management *(responses obtained using a 7-point Likert-type scale where 1 = completely unaware and 7 = strongly agree);*
- knowledge of ISO 9000 standards *(responses obtained using a 7-point Likert-type scale where 1 = completely unaware and 7 = strongly agree);*
- proportion of staff to have attended training in quality management *(responses obtained using a 7-point Likert-type scale where 1 = nobody and 7 = everybody;*
- administrative staff competence in quality management *(responses obtained using a 7-point Likert-type scale where 1 = very low and 7 = high);*
- nursing staff competence in quality management *(responses obtained using a 7-point Likert-type scale where 1 = very low and 7 = high);*
- doctors’ competence in quality management *(responses obtained using a 7-point Likert-type scale where 1 = very low and 7 = high);*
- administrative staff competence in quality management *(responses obtained using a 7-point Likert-type scale where 1 = very low and 7 = high);*
- other staff competence in quality management *(responses obtained using a 7-point Likert-type scale where 1 = very low and 7 = high);*
- existence of a formal audit group *(yes/no/not aware);*
- existence of a local audit group manager *(yes/no);*
- current QMS implementation stage *(functioning/under implementation/absent/other);*
- existence of a system for staff training in quality management *(yes/no/partly);*

**Questions on QMS implementation problems:**

*(responses on all below items obtained using a 7-point Likert-type scale where 1 = strongly disagree and 7 = strongly agree)*

- quality policy definition;
- quality goal setting;
- appointment of a local audit group manager;
- formation of a local audit group;
- staff training;
- procedure description;
- work procedure formalization;
- usage of diagnostic and treatment methods;
- employee resistance to the QMS implementation;
- lack of information;
- lack of financial resources;

**Questions on QMS benefits and level of managerial satisfaction with the operating QMS:**

- QMS implementation benefits for the organization: *(responses on all below items obtained using a 7-point Likert-type scale where 1 = strongly disagree and 7 = strongly agree):*
- better financial situation;
- improved service quality;
- higher customer satisfaction;
- more effective communication;
- higher staff motivation;
- improved patient safety;
- lower rate of undesirable incidents;
- increased patient number;
- fewer mistakes;
- better work organization;
- safer work environment;
- better employee interrelationship;
- better responsibility and authority distribution;
- satisfaction with the operating QMS *(responses obtained using a 7-point Likert-type scale where 1 = completely dissatisfied and 7 = completely satisfied);*
- perceived QMS significance for the institution *(responses obtained using a 7-point Likert-type scale where 1 = completely irrelevant and 7 = completely relevant).*
